# Coupling Between Cell Cycle Progression and the Nuclear RNA Polymerases System

**DOI:** 10.3389/fmolb.2021.691636

**Published:** 2021-08-02

**Authors:** Irene Delgado-Román, Mari Cruz Muñoz-Centeno

**Affiliations:** ^1^ Instituto de Biomedicina de Sevilla, Universidad de Sevilla-CSIC-Hospital Universitario V. Del Rocío, Seville, Spain; ^2^ Departamento de Genética, Facultad de Biología, Universidad de Sevilla, Seville, Spain

**Keywords:** RNA polymerases I, II and III, RNA polymerases assembly, cell cycle progression, regulatory networks, *Sacharomyces cerevisiae*

## Abstract

Eukaryotic life is possible due to the multitude of complex and precise phenomena that take place in the cell. Essential processes like gene transcription, mRNA translation, cell growth, and proliferation, or membrane traffic, among many others, are strictly regulated to ensure functional success. Such systems or vital processes do not work and adjusts independently of each other. It is required to ensure coordination among them which requires communication, or crosstalk, between their different elements through the establishment of complex regulatory networks. Distortion of this coordination affects, not only the specific processes involved, but also the whole cell fate. However, the connection between some systems and cell fate, is not yet very well understood and opens lots of interesting questions. In this review, we focus on the coordination between the function of the three nuclear RNA polymerases and cell cycle progression. Although we mainly focus on the model organism *Saccharomyces cerevisiae*, different aspects and similarities in higher eukaryotes are also addressed. We will first focus on how the different phases of the cell cycle affect the RNA polymerases activity and then how RNA polymerases status impacts on cell cycle. A good example of how RNA polymerases functions impact on cell cycle is the ribosome biogenesis process, which needs the coordinated and balanced production of mRNAs and rRNAs synthesized by the three eukaryotic RNA polymerases. Distortions of this balance generates ribosome biogenesis alterations that can impact cell cycle progression. We also pay attention to those cases where specific cell cycle defects generate in response to repressed synthesis of ribosomal proteins or RNA polymerases assembly defects.

## Introduction

The eukaryotic cell cycle is controlled by a regulatory network, whose general features are conserved from yeast to humans (Lubischer, 2007). It proceeds through firmly regulated transitions to ensure that specific events take place in a correct and organized manner. This, in turn, ensures viability and the correct transmission of genetic information (Haase and Wittenberg, 2014). A fundamental element of cell cycle regulation consists of arrests at particular steps to guarantee the completion of a previous cell cycle event, to repair cellular or DNA damage, or to resolve a challenging situation. Accordingly, eukaryotic cell cycle regulation integrates a huge multitude of internal and external signals to optimize survival. Failures in these processes reduce cell survival and, in higher metazoans, lead to cancer, and other diseases (Moriel-Carretero et al., 2019; She et al., 2019; Klemm et al., 2020; Lai et al., 2020; Matellán and Monje-Casas, 2020; Niwa, 2020).

RNA synthesis in the eukaryotic nucleus is carried out by three multisubunit complexes. RNA polymerase II (RNA pol II) transcribes the vast majority of genes, including all protein coding and many other non-coding RNAs (ncRNAs) such as snRNAs, miRNAs, and snoRNAs. RNA polymerase I (RNA pol I) transcribes ribosomal RNAs (rRNA) as a single polycistronic gene: rRNA 35-47S, which is processed into 3 mature rRNAs: 28S (25S in yeast), 18S and 5.8S. This gene appears repeatedly in all eukaryotes with hundreds of copies arranged in tandem. RNA polymerase III (RNA pol III) transcribes an intermediate number of small, non-coding genes (150–400 different), including 5S rRNA and tRNAs (Chan and Lowe, 2016). RNA pol I transcription accounts for almost 60% of global transcription and RNA pol III for around 25%. Of the latter, the 5S rRNA constitutes between 10–15%; and the rest, mostly corresponds to tRNAs. Finally, RNA pol II transcription corresponds to approximately 15% of the total. An important part of this corresponds to RNAs that encode ribosomal proteins (Warner, 1999; Pelechano et al., 2010).

The connection between this transcriptional network and cell cycle progression, can be divided into two different aspects with different levels of knowledge. Regarding what we can call “better known word of the RNA polymerases and the cell cycle,” lot of information has been generated describing the dramatic reorganization of gene expression that takes place through the cell cycle. Nearly 20% of *S. cerevisiae* yeast genome is transcribed periodically during each cell division cycle. Abundant information is available on the waves of genes expression associated to the different phases (G1, S, G2/M, M/G1), the complex regulatory connection between them, and on the technological approaches to study this phenomenon (Haase and Wittenberg, 2014). Obviously, in this better-known world, we can understand how transcription impairment of specific genes can disturb the normal cell cycle progression. At this level, RNA pol II has a relevant and direct role on cell cycle regulation (Hartwell et al., 1973; Bähler, 2005; Nurse, 2020).

In this review, we focus in the “lesser known world of the RNA polymerases and the cell cycle.” During years, there has been an increase in the knowledge of connections between complex regulatory networks as the transcriptional machinery and cell cycle progression. General changes on transcription levels depending on the cell cycle phases has been known for over decades (Gottesfeld and Forbes, 1997). Different biochemical events underlying this coupled regulation have been elucidated. Here we will focus on mechanisms affecting the three nuclear RNA polymerases. Here we also address this crosstalk between cell cycle and RNA polymerases in the opposite sense, that is, how the status and function of nuclear RNA polymerases can affect cell cycle progression, a much lesser known aspect. It is important to highlight that this interplay coordinates different aspects of the overall status of the three polymerases system with cell cycle progression. In this sense, we review how cell cycle regulation is affected by the balance between the three RNA polymerases products and, secondly, by RNA polymerases assembly. Finally, we also analyze the parallelism between these regulatory interplays in yeast and metazoan, suggesting that it could exist a general control strategy extended throughout eukaryotes.

## Cell Cycle Phases Impacts on RNA Polymerases Function

Since several years, it is well known that transcription activity in eukaryotes is affected by cell cycle phases. Thus, transcription is repressed during mitosis and highly active in interphase (G1, S, and G2). This mitotic repression has been observed *in vivo* for genes transcribed by all three nuclear RNA polymerases. Different mechanisms contribute to mitotic repression as global transcriptional silencing, including dissociation of transcription factors and cofactors from target genes and profound reorganization of chromatin structure (Gottesfeld and Forbes, 1997, and references therein; Taylor, 1960; Marsden and Laemmli, 1979; Martínez-Balbás et al., 1995). We will focus on how cell cycle phases modulate transcription affecting the basal transcription machinery (RNA pol I, II, and II) in *S. cerevisiae* although some aspects in higher eukaryotes will also be addressed.

### RNA Pol II Transcribing Through the Cell Phases

Early works, interestingly described a cell cycle arrest for some RNA pol II mutants. Thus, mutations in the largest RNA pol II subunit, Rpb1, impaired cell cycle progression in budding yeast *S. cerevisiae* (Drebot et al., 1993), fission yeast *Schizosaccharomyces pombe* (Sugaya et al., 1998) and mammalian cells (Sugaya et al., 2001). RNA pol II activity is regulated during the cell cycle by changes in the phosphorylation status of the carboxyl-terminal domain (CTD) of its largest subunit Rpb1 both in yeast and mammal cells (Bregman et al., 2000; Oelgeschläger, 2002; Chymkowitch and Enserink, 2013). The Rpb1 CTD contains 26 heptapeptide repeats in yeast (Allison et al., 1988) and 52 in mammals (Corden et al., 1985). The direct regulation of CTD phosphorylation serves as a switch to regulate transcription machinery during the cell cycle. In the budding yeast *S. cerevisiae*, early in the transcription cycle, Kin28 phosphorylates the CTD which serves as a mark for recruitment of the mRNA capping system (Rodriguez et al., 2000). Interestingly, and coupling cell cycle to RNA pol II activity, it has been demonstrated that Cdc28 (also called Cdk1, and the main CDK cell cycle regulator in budding yeast) is a CTD kinase sharing a partially redundant role with Kin28 (Chymkowitch et al., 2012; Chymkowitch and Enserink, 2013).

### RNA Pol III Transcribing Through the Cell Phases

A tRNA synthesis fluctuation during cell cycle has been described both in mammals and yeast (Scott et al., 2001; Frenkel-Morgenstern et al., 2012; Chen and Gartenberg, 2014; Herrera et al., 2018). Previous results had proposed a tRNA peak in M phase (Chen and Gartenberg, 2014). However, a more recent research has demonstrated that *tDNA* transcription peaked in S phase. The authors, interestingly, propose that this apparent discrepancy can be explained by the overlapping between the S phase and metaphase in *S. cerevisiae*, concluding that the cell cycle-dependent increase in tDNA transcription occurs in the overlapping time span of late S phase/early metaphase. The same authors demonstrate the regulatory mechanism coupling cell cycle to RNA pol III activity: the S phase cyclin Clb5 recruits Cdc28 (Cdk1) to *tDNA* genes; Cdc28 promotes the recruitment of TFIIIC and stimulates the interaction TFIIIC/TFIIIB which directly increases the dynamics of RNA pol III *in vivo*. Bdp1, a component of the TFIIIB complex, has been proposed as the direct target for Cdc28 (Herrera et al., 2018). Recently, new post-translational modifications of RNA pol III, as sumoylation, has been proposed to be involved in stress response in yeast (Nguéa P et al., 2019). The role of this modifications in cell cycle would also be a very interesting open question.

### RNA Pol I Transcribing Through the Cell Phases

Transcription by RNA pol I oscillates during the cell cycle, being repressed during mitosis, recovered during G1 and maximal in S/G2 phases. In mammals, repression during M phase is caused by inactivation of a RNA pol I specific factor (TIF-IB/SL1) by an inhibitory cdc2 mediated phosphorylation (Heix et al., 1998). Then, transcription recovery during G1 is mediated by reactivation of another specific factor, UBF (Klein and Grummt, 1999). In the budding yeast, the locus containing *rDNA* genes, segregate after the rest of the genome, in late anaphase. Only in anaphase, yeast repress RNA pol I transcription by the Cdc14 phosphatase acting on Rpa43 subunit, inducing the dissociation of RNA pol I from the 35S *rDNA* (Clemente-Blanco et al., 2009). More recently in *S. cerevisiae*, it has been demonstrated that Rio1 downregulates RNA pol I in a cell cycle dependent manner through Rpa43 subunit as a target. Moreover, Rio1 promotes rDNA stability to ensure rDNA segregation during anaphase (Iacovella et al., 2015).

## Imbalance of RNA Pol I, II, and III Products Provokes G1 Arrest

### Balanced Production of Ribosomal Components Prevents G1 Arrest in Budding Yeast

NTP-depleting drugs, as 6-Azauracil (6AU) and mycophenolic acid (MPA) interfere with transcription elongation *in vivo* by strongly inhibiting inosine monophosfate (IMP) dehydrogenase, a rate-limiting enzyme in the *novo* synthesis in guanine nucleotides (Shaw and Reines, 2000; Shaw et al., 2001). Our studies revealed that *S. cerevisiae* cells accumulate at G1 after NTP-depleting drug treatment. As NTP are substrates for three RNA polymerases, we could clearly establish that NTP depletion differentially impacts the RNA products of the three RNA polymerases: products from RNA pol I and III presented a strong and early reduction after treatment but mRNAs showed a very slight reduction at the same conditions. Thus, NTP-depletion drugs generate a clear imbalance between pre-rRNAs, tRNAs and mRNAs (Gómez-Herreros et al., 2013). Using conditional mutants affecting essential subunits of RNA pol I (Rpa43) or III (Rpc17), where their normal transcripts production (rRNAs or 5S rRNA respectively) decreased but not mRNAs generated from wild type RNA pol II, cells also arrested at G1, indicating that any imbalance in RNA polymerases products negatively impacts G1/S transition (Gómez-Herreros et al., 2013).

**FIGURE 1:**
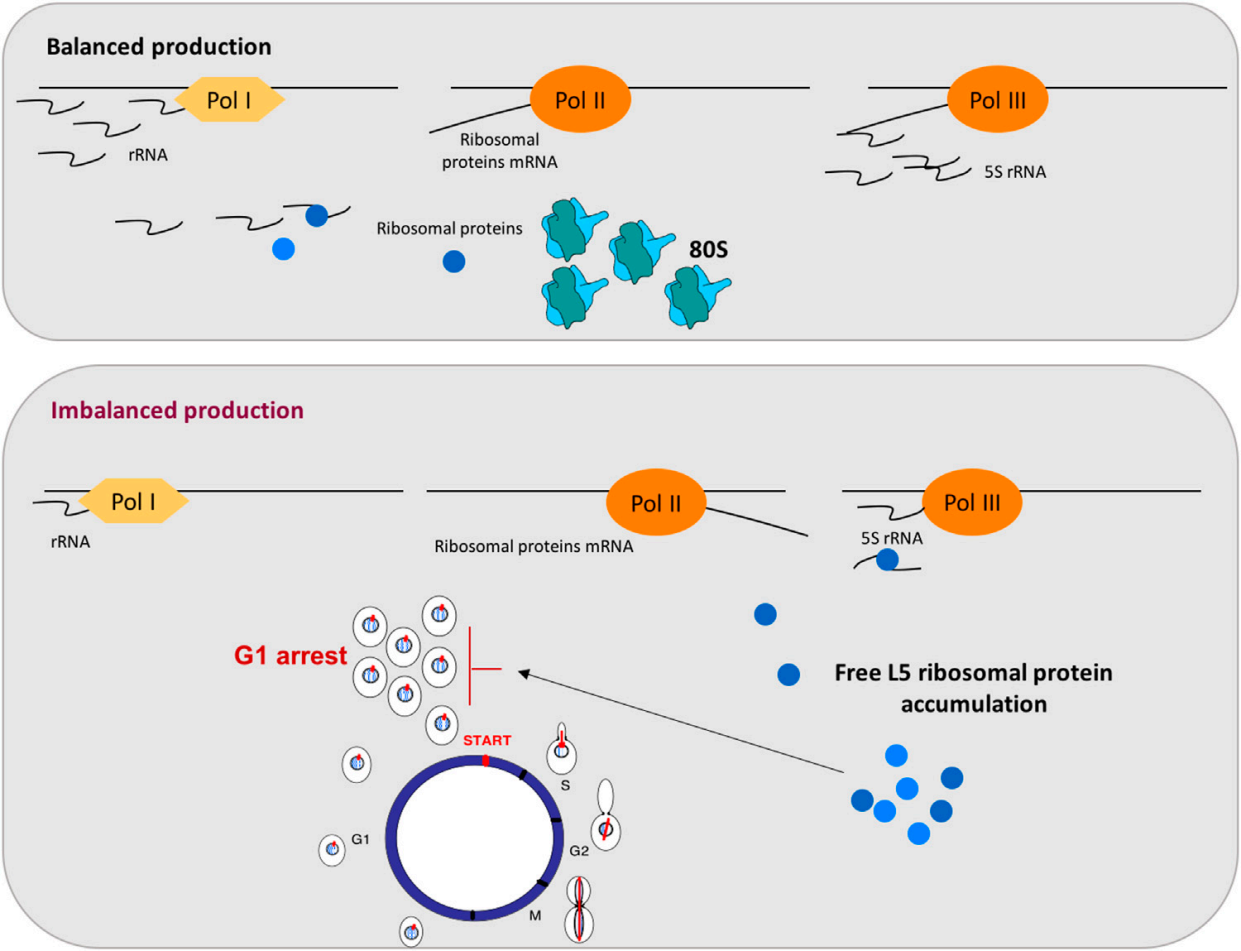
Coupling RNA polymerases production to cell cycle through the free accumulation of the r-protein L5 in yeast. The top panel represents balanced production of ribosomal components: rRNA, r-proteins mRNA and 5S rRNA, transcribed by RNA pol I, II, and III, respectively, are synthesized in the balanced proportion required for correct ribosomal particles assembly. The bottom panel represents situations where this balance is disturbed by a decrease in rRNAs levels but not in r-proteins mRNAs, generating free L5 accumulation and a G1 arrest. As indicated in the figure, rRNA is represented by waves and mRNA by lines.

Ribosome biogenesis is a highly resource-consuming process and, therefore, involves the tight regulation and balanced synthesis of all its components. This complicated pathway requires the coordinated assembly of rRNAs, synthesized by RNA pol I and III, and ribosomal proteins (r-proteins), whose mRNAs are transcribed by RNA pol II. This coordination is critical for an effective utilization of cell resources and requires a balanced function of the RNA pol I, II, and III transcription activities. Thus, the synthesis of rRNAs and r-proteins are two coordinated pathways that lead to efficient ribosome biogenesis [(Warner, 1999; de la Cruz et al., 2018) and references therein]. Data from mammalian cells also showed a G1 arrest after disturbances in ribosome biogenesis, moreover, a key role of mammalian r-proteins L5 and L11 for this essential response has been demonstrated very well (Sun et al., 2008). L11 and L5 r-proteins assembly to 5S rRNA on pre-60S ribosomal particles in a process mediated by Rrs1 (Miyoshi et al., 2004). These mammalian r-proteins L5 and L11 have been reported to accumulate as free proteins and to induce p53 stabilization and G1 arrest after ribosomal biogenesis stress (Sun et al., 2008; Bursać et al., 2012). Therefore, we proposed that in yeast, the imbalance in the three RNA polymerases transcripts provoked defects in ribosomal biogenesis and generated the accumulation of free r-proteins due to the drop in rRNAs. This ribosomal assembly defect could induce a G1 arrest through the accumulation of free r-proteins. Thus, we demonstrated the accumulation of free L5 r-protein in these conditions, as was the case for mammalian cells. Figure 1 summarizes the model that has been proposed (Gómez-Herreros et al., 2013). In this model, the balanced activity of the three eukaryotic RNA polymerases (I, II, and III) is a prerequisite for an equimolar production of the different ribosomal components. When this balance is disturbed, the accumulation of free L5 occurs and acts as a signal to arrest cell cycle at G1 (Figure 1).

Specific cell cycle defects have been described in response to repressed synthesis of r-proteins. After several hours of repression of r-proteins, systematic analyses of cell cycle progression, cell morphology, and bud site selection were performed after repression of 54 individual r-proteins genes in *S. cerevisiae*. In this study, most of the repressed genes involved a G1 arrest (nine encoding 60S subunit components and twenty-two encoding r-proteins of the 40S subunit) and only nine repressed genes encoding components of the 60S subunit resulted in a G2/M delay (Thapa et al., 2013). A later work from the same laboratory, explore cell cycle changes during the transition from normal cell cycle to arrest after inhibition of ribosome formation or translation capacity. Both inhibitions are sensed after a short time and the G1 stage was reached. No spindles or mitotic actin rings were visible, but membrane ingression was completed in most cells and Ace2, a transcription factor with asymmetric localization to daughter cell nuclei after cell division (Herrero et al., 2020), was localized to daughter cell nuclei demonstrating that, even in the budded arrested cells, G1 phase was reached (Shamsuzzaman et al., 2017). Finally, and very recently, it has been shown that disruption of the assembly of the 40S subunit affected the assembly of the 60S subunit (Rahman et al., 2020). As the r-proteins in each ribosomal subunit are essential only for the assembly of the cognate subunit (Gregory et al., 2019), it was unexpected that disruption of the 40S subunit assembly affected the kinetics of assembly of the 60S subunit, causing accumulation of free/extra-ribosomal 60S L5 (also named uL8) (Rahman et al., 2020). These results indicate that an interaction between the assembly of ribosomal subunits 40S and 60S exists, and that free L5 is a good marker of this generated ribosomal stress.

### Nucleolar Stress Induces a G1 Arrest in Mammalian Cells

Nucleolar stress is the term used to described failures in ribosome biogenesis or function that ultimately leads to disruption in cell homeostasis (James et al., 2014). In human cells, mycophenolic acid (MPA) acts as an NTP-depleting drug, as in yeast. Thus, in mammalian, MPA treatment results in both a drastic reduction of pre-rRNA synthesis and the disruption of the nucleolus, causing p53 activation and the subsequent G1 arrest. This treatment provokes the accumulation of free human r-proteins L5 and L11 that bind and inhibit MDM2, the p53 E3 ubiquitin ligase. Therefore, ribosomal imbalance causes MDM2 inhibition, which induces p53 stabilization (Sun et al., 2008; Bursać et al., 2012; Fumagalli et al., 2012; Lo et al., 2012).

Cell responses to the imbalance between RNA polymerases activities, described in yeast and human cells, show very strong analogies: i) in both systems the outcome is a G1 arrest; ii) in both organisms, the G1 arrest responses are mediated by a ribosomal stress; iii) in both scenarios the accumulation of free r-proteins (as L5) is essential for coupling to cell cycle. This strong parallelism between the mechanisms responding to nucleolar stress in yeast and metazoan suggests that it reflects a general control strategy extended throughout eukaryotes. However, a major difference between the two systems exists: yeast does not contain p53 or MDM2. The interpretation of these differences has been extensively discussed and other systems exhibiting nucleolar stress without p53 have been described (James et al., 2014).

## Deffects in RNA Polymerase Assembly Provokes Arrest at G1

As we have just described, the ribosome biogenesis process has been extensively studied [(de la Cruz et al., 2018) and references therein] and its relevant role in interplaying complex networks, as cell cycle regulation, has been revealed. The assembly of eukaryotic RNA polymerases (RNA pol I, II, and III), is not completely understood although some elements involved in that process has been recently identified. We focus on yeast RNA pol III assembly, as coupling between this assembly process and cell cycle progression has been described (Płonka et al., 2019). The authors had previously isolated and characterized conditional mutants affecting the Rpc128, the second largest RNA pol III subunit. The mutant allele *rpc128-1007* presents a severe defect in RNA pol III assembly as well as an expected reduction in tRNA levels (Cieśla et al., 2007; Cieśla et al., 2015). This conditional mutant, at the restrictive temperature, shows a G1 arrest phenotype which is partially suppressed by overexpression of *RBS1*, the gene encoding a protein involved in RNA pol III assembly (Cieśla et al., 2015). Also, cells lacking Rbs1 showed moderated delay in G1/S transition, indicating that impaired RNA pol III assembly is connected to the cell cycle default. Moreover, the G1 arrest phenotype is not suppressed after inactivation of Maf1, conditions in which elevated levels of tRNAs are produced (Pluta et al., 2001). Thus, they conclude that impairment of RNA pol III complex assembly, and not decreased tRNA transcription levels, is the primary reason for the G1 arrest observed in the rpc128 mutant (Płonka et al., 2019). Very interestingly, Rbs1 was identified as a substrate of cyclin-dependent kinase Cdc28, the main cell cycle regulator in *S. cerevisiae*, in a global proteomic approach (Ubersax et al., 2003).

However, there is evidence that RNA pol III defects can affect cell cycle progression regardless of assembly defects. Thus, mutants affecting the Rpc53 RNA pol III subunit, which has not been described as involved in assembly, leads to a G1 arrest both in yeast (Mann et al., 1992) and mammals (Ittmann et al., 1993). Moreover, depletion of *RPC17* (encoding another RNA pol III subunit), also led to a delay in the G1 phase of the cell cycle (Gómez-Herreros et al., 2013) but, interestingly, *RBS1* overexpression did not overcome G1 arrest (Płonka et al., 2019). These results indicate that G1 arrest coupled to defects in RNA Pol III can be mediated by different regulatory inputs.

## Conclusions, Applications and Open Questions

**FIGURE 2:**
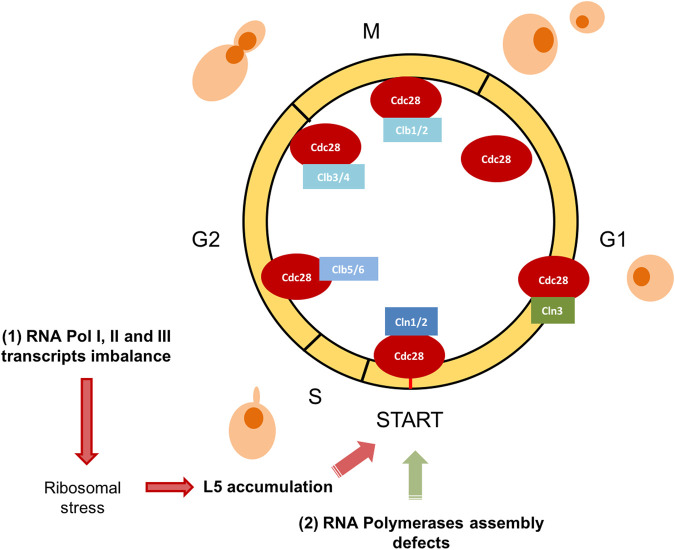
*S. cerevisiae* yeast cell cycle scheme. The RNA polymerases system status impacts on G1/S transition. Graphical representation of the cell cycle phases and main regulators in *S. cerevisiae* are designed. The cyclin-dependent kinase Cdc28 (also known as Cdk1) is sufficient and necessary for cell cycle regulation. Different substrates are phosphorylated based on their association with G1 phase cyclins (Cln1, 2, and 3), S phase cyclins (Clb 5 and 6) or mitotic cyclins (Clb 1, 2, 3, and 4). In this figure, we represent how two abnormal situations involving RNA polymerases, affects specifically G1/S transition: (1) RNA pol I, II, and III transcripts imbalance and (2) RNA polymerases assembly defects.

In this work, we have revisited some aspects of the crosstalk between cell cycle progression and RNA polymerases function. We have focused on those situations where the cell cycle defect is not mediated by the limiting transcription of a specific gene, but those situations where the signal for the cell cycle regulation is the consequence of impaired activity of RNA polymerases or this activity is modulated by the cell cycle phase. First, we have revisited how the three RNA polymerases modulates their transcription capacity by cell cycle. Then, we have discussed two models in yeast. The first one, when cell cycle arrest is generated by an imbalanced production of RNA pol I, II, and III, which induces an imbalance in ribosomal components and the accumulation of the free r-protein L5 (Figure 1). Secondly, when a defect in RNA polymerases assembly is sensed and cell cycle arrested. In both cases, cells arrest at G1, indicating that yeast cells are able to detect internal signals, derived from the activity of the transcriptional machinery. These signals can impact the dynamics of START, the main regulatory event that takes place towards the end of G1 and involves an extensive transcriptional program (Costanzo et al., 2004; de Bruin et al., 2004; Haase and Wittenberg, 2014). It is a very attractive concept that complex processes like gene transcription and ribosomal biogenesis are coupled and sensed to take decisions at START (Figure 2).

We have also highlighted that the surveillance mechanism that couples balanced production of yeast ribosomal components and cell cycle, resembles the p53-dependent nucleolar stress checkpoint described in human cells, which indicates that this is a general control strategy extended throughout eukaryotes. In human cells, the molecular components of the regulatory pathway are well known. Clinicians uses the induction of nucleolar stress in cancer cells as an anti-cancer therapy. Moreover, selective inhibition of ribosomal gene transcription in the nucleolus has been shown to be an effective therapeutic strategy to promote cancer-specific activation of p53 (Bywater et al., 2012; Hein et al., 2013; James et al., 2014; Woods et al., 2015; Carotenuto et al., 2019).

Relevant questions remain to be answered in the yeast regulatory systems presented in this work. First, it would be interesting to figure out if all G1 arrest phenomena induced by different defects in RNA polymerases are mediated by the ribosomal stress. Finally, it would be extremely challenging to elucidate the molecular elements that connect the signals (imbalanced production of ribosomal components or defects in assembly) to the G1 arrest. The different elements that participate in the G1/S transition regulatory network, are good candidates. This knowledge would have a relevant translational potential as more than 50% of human cancers lack functional p53. Identification of new p53-independent response pathways could potentially reveal new therapy strategies for p53-defective cancer.

In summary, only understanding both regulatory aspects of this crosstalk, how cell cycle modulates transcription and *viceversa*, a precise knowledge of this complex regulatory interplay will be achieved with a huge translational potential that it has already begun promisingly.
